# A Transcriptomic Study on the Toxic Effects of Iodide (I^−^) Wet Deposition on Pepper (*Capsicum annuum*) Leaves

**DOI:** 10.3390/cimb47050313

**Published:** 2025-04-28

**Authors:** Rui Yu, Zhu-Ling Ma, Min Wang, Jie Jin

**Affiliations:** School of Environmental Science and Engineering, North China Electric Power University, Beijing 102206, China; 15732503913@163.com (R.Y.); zhuling_ma@163.com (Z.-L.M.); mm17735579126@163.com (M.W.)

**Keywords:** wet-deposited iodine, *Capsicum annuum* L., transcriptomics, differential gene expression, functional enrichment analysis, toxic effects

## Abstract

Radioactive iodine (^129^I), released into the environment from human nuclear activities, poses significant health risks to the biosphere due to its long half-life and mobility. This study investigates the toxic effects of wet-deposited iodine on the growth of chili pepper seedlings (*Capsicum annuum* L.) under soil cultivation conditions. Using sodium iodide (NaI) as the exposure agent, transcriptomic analysis was conducted to evaluate the molecular responses of chili pepper leaves to iodine at concentrations of 2, 4, and 8 ppm. The study identified 2440 and 1543 differentially expressed genes (DEGs) in leaves exposed to 2 ppm vs. 4 ppm iodine and 2 ppm vs. 8 ppm iodine, respectively. GO enrichment analysis showed that DEGs at 4 ppm were significantly associated with protein–chromophore linkage, extracellular region, and iron ion binding, while those at 8 ppm were enriched in defense response, cell wall components, and iron ion binding. Iodine stress disrupted key pathways associated with photosynthesis, antioxidant defense, and cuticle biosynthesis. In particular, the downregulation of key genes related to protein–chromophore binding, lipid metabolism, and cell wall organization indicated reduced photosynthetic efficiency and weakened stress resistance. This study provides molecular-level insights into the ecological risks of iodine stress in plants and offers a scientific basis for managing iodine contamination and breeding iodine-tolerant chili pepper cultivars.

## 1. Introduction

With the continuous growth of global energy demand and the rapid development of nuclear technology, the effective management of spent nuclear fuel has become one of the critical challenges to address [1]. During the depletion treatment of spent nuclear fuel, gaseous effluents containing radioactive isotope ^129^I are inevitably produced [2]. Given the high volatility, long half-life, and relatively strong radiotoxicity of ^129^I, it accumulates in the environment over time and exhibits biomagnification, posing a significant threat to ecological balance and human health [3]. As a fundamental component of ecosystems, plants are a key link in the entry of radioactive substances into the food chain and serve as important indicators for the ecological risk assessment of radioactive iodine [4].

A substantial body of scientific research has shown that trace amounts of iodine play an indispensable role in plant growth and development. In the study by Kiferle et al. [5], ^125^I was used as a nutrient source, and SDS-PAGE analysis of *Arabidopsis thaliana* protein extracts revealed, for the first time, the binding of iodine to proteins in the plant roots and shoots. The study also demonstrated that iodinated proteins in the stem were class III peroxidases (PODs), which participate in and enhance the plant’s photosynthetic process, thus promoting the efficient synthesis of carbohydrates and proteins within the plant. Additionally, iodine functions as an efficient antioxidant in plants [6], and as iodine content increases, the plant’s antioxidant capacity is also enhanced [7]. This is likely because reactive oxygen species (ROS), metabolic by-products produced during photosynthesis, can be eliminated by PODs, enabling plant cells to resist oxidative stress and maintain physiological stability. Furthermore, low levels of iodine can effectively enhance the plant’s stress tolerance [8], aiding in resistance to both biotic and abiotic stresses [9].

However, excessive accumulation of iodine can have detrimental effects on plant growth [10]. For instance, rice blast disease observed in low-lying rice fields in Japan has been linked to iodine toxicity caused by excessive iodine accumulation [11]. Iodine toxicity primarily affects the physiological and biochemical activities of plants by inducing the production of excess ROS, which disrupts redox homeostasis and leads to an oxidative stress state [5]. This imbalance can damage plant cell structures, causing functional disorders (such as enzyme inactivation and lipid peroxidation) and, in severe cases, cell death. Excess iodine also reduces chlorophyll content in plants [12], adversely affecting photosynthesis.

*Capsicum annuum* L., commonly known as chili pepper, has garnered considerable attention due to its rich nutritional value and good environmental adaptability. The various bioactive molecules it contains, such as ascorbic acid, sugars, polyphenols, carotenoids, and antioxidants [13], contribute to enhanced immune function, resistance to oxidative stress, and reduced risk of chronic diseases, thus promoting human health. Notably, chili peppers have a high capacity for iodine absorption and accumulation [14]. Research has shown that plants mainly accumulate iodine from the environment through two primary pathways: first, by absorbing iodine from the soil via their well-developed root systems, and second, by capturing iodine from the air through their leaves [15]. Studies have suggested that plant leaves may have superior efficiency in absorbing exogenous iodine compared to traditional root absorption [16,17]. Studies have shown that iodide ions are one of the primary forms of iodine in wet deposition [18,19] and, also, that the environmental behavior of radioactive iodine is similar to that of non-radioactive iodine, exhibiting both bioaffinity and significant environmental mobility [20,21]. Based on this characteristic, this study employs non-radioactive iodine (NaI) as a surrogate for radioactive iodine to investigate its translocation and transformation in plants. The findings will provide a theoretical basis for assessing the ecological risks posed by radioactive iodine.

Therefore, this study focuses on the toxicity effects of wet-deposited iodine on the leaves of chili pepper seedlings under soil cultivation conditions. Sodium iodide (NaI) was chosen as the experimental exposure substance to explore the toxic effects of iodine deposition on chili pepper leaves.

## 2. Materials and Methods

### 2.1. Cultivation of Chili Peppers

The study employed line pepper (*Capsicum annuum* L.) as the model plant. Specifically, we used Erjingtiao, a representative chili pepper cultivar from Sichuan Province, China. Seeds were procured from the Beijing Academy of Agriculture and Forestry Sciences. *Capsicum annuum* L. healthy, intact, and plump chili pepper seeds were randomly selected for the experiment. Before the experiment, the seeds were thoroughly cleaned and soaked in a 1% bleach solution (NaClO, Merck, Darmstadt, Germany, analytical grade ≥ 99.99%) for several minutes, followed by a thorough rinse with ionized water to remove any residual impurities. The treated seeds were then soaked in warm water (approximately 25–32 °C) for 24 h to accelerate the germination process. After soaking, the seeds were placed between two layers of wet paper towels and kept in a warm environment (such as 20–25 °C) in the dark to improve germination rates and shorten the germination period. During this phase, the paper towels needed to remain moist, but without excess water accumulation. The optimal germination temperature for chili pepper seeds is around 25–30 °C, so a heating pad or a constant-temperature indoor environment may be used to maintain this temperature.

During germination, seeds were regularly monitored for fungal contamination or other pathogen infections. As soon as the capsicum (*Capsicum annuum*) radicle broke through the seed coat, the germinated seeds were transferred to a controlled-environment growth chamber (model: MRC-1100D-LED; Prante, Jinhua, China) with the light intensity set at 10,000 lux. When the seedlings developed one to two pairs of true leaves (excluding cotyledons), they were transplanted into 15-cm-diameter plastic pots (one plant per pot) with three biological replicates per treatment group. The pots were filled with surface (0–20 cm) purple soil (Clay Loam) collected from Sichuan Province, China (31°20′ N, 105°56′ E). Plants were cultivated under the following controlled conditions: (1) light intensity: 10,000 Lux (12 h/12 h light/dark cycle); (2) day/night temperature: 25 ± 1 °C (light period)/18 ± 1 °C (dark period); (3) relative humidity: 60–70%; and (4) watering: 70 mL deionized water per pot daily. This stage typically occurred 3–4 weeks post-sowing, though exact timing varied with growth conditions.

### 2.2. Wet Deposition Iodine Exposure Experiment

**Figure 1:**
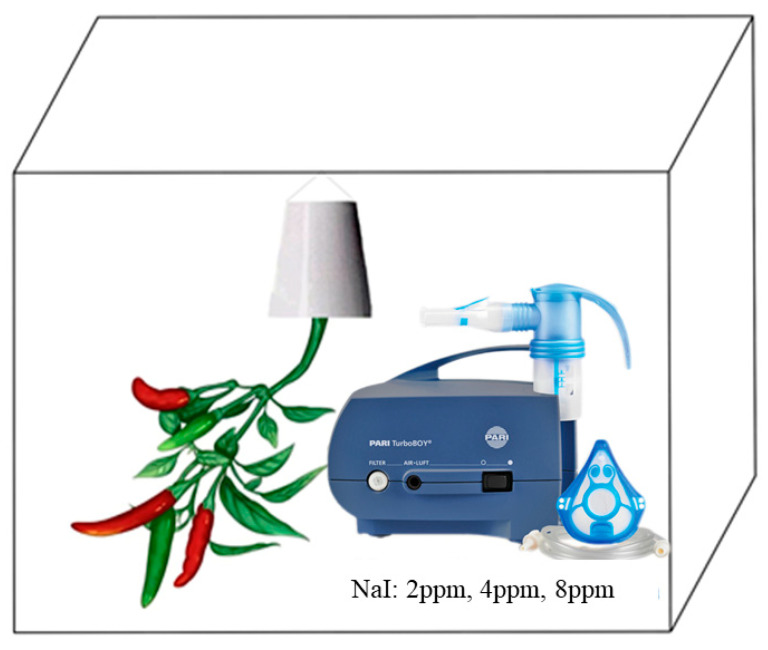
A conceptual diagram of the wet deposition iodine exposure experiment.

Once the chili pepper plants bore fruit approximately 1–2 cm in length, they were placed in a pre-constructed closed growth chamber (composed of a lidless storage box and a plastic wrap-covered top, as shown in Figure 1), with precise control over environmental parameters such as temperature, humidity, and light intensity to ensure optimal plant growth and development.

The inverted growth mode was chosen for two main reasons: First, due to the limited availability of soil resources, when the soil moisture reaches saturation, the plant roots often exhibit a floating growth pattern at the soil surface, which significantly weakens the plant’s ability to absorb nutrients from the soil. The inverted growth mode increases the root–soil contact area, effectively avoiding this issue and providing a superior environment for plant growth. Secondly, the inverted growth pattern also constructs a unique research environment, which not only promotes the growth and development of plant leaves [22,23] but also enables the plant itself to be in full contact with the gaseous iodine atmosphere only, which skillfully excludes the possibility of external iodine elements being absorbed by the plant roots through the soil and improves the accuracy of the experiment.

Translated with DeepL.com (free version) to generate specific iodide (I^−^) exposure regimes, an ultrasonic precision nebulizer (TurboBOY N085G3255, PARI, Starnberg, Germany) was utilized to atomize aqueous solutions of sodium iodide (NaI, Merck, analytical grade ≥ 99.99%), ensuring that the chili pepper leaves were evenly exposed to the iodine ion environment. The exposure experiment consisted of three groups with iodine concentrations of 2 ppm, 4 ppm, and 8 ppm I^−^, labeled from low to high concentration as PEYP-1-1, PEYP-1-2, and PEYP-1-3, respectively. For each biological replicate, three mature leaves (fully expanded, 4th–6th nodal position) per treatment were harvested, immediately flash-frozen in liquid N_2_, and maintained at −80 °C until RNA extraction for transcriptomic sequencing. A quantitative assessment based on spatial volume occupancy showed that the aboveground biomass of a single wire pepper plant was approximately 12.5% (1/8) of the effective volume of the growth chamber. According to the ideal gas distribution model, under the assumption of homogeneous distribution of iodine ions in a closed system, 12.5% of the applied amount could theoretically be in gas–solid phase contact with plant foliage, i.e., the actual iodine contact concentrations of PEYP-1-1, PEYP-1-2, and PEYP-1-3 were 0.25 ppm, 0.5 ppm, and 1 ppm, respectively.

The control group for the chili pepper leaves was exposed to 2 ppm iodine rather than being a blank control group, as Li et al. [24] found that, under conditions of 0.25 mg/L I^−^, the appearance of the leaves, chlorophyll content, and the activity of various enzymes were more optimal than in the absence of iodine.

Therefore, 2 ppm I^−^ was chosen as the control group in this experiment to facilitate more direct observation of iodine’s toxic effects on chili pepper leaves.

The exposure experiment lasted for 15 days, during which environmental parameters were regularly monitored and adjusted to maintain optimal growth conditions for the plants, ensuring continuous stability of the experimental conditions.

### 2.3. Transcriptome Sequencing

1. RNA extraction (TRIzol method): Total RNA was extracted using a TRIzol kit (Thermo Fisher Scientific, Waltham, MA, USA). Briefly, tissue samples were milled in liquid nitrogen and then fully lysed by adding TRIzol and centrifuged to remove impurities. Chloroform (CHCl_3_, Merck, ≥99.5%) was added for phase separation, and isopropanol (C_3_H_8_O, Merck, ≥99.9%) was used to precipitate RNA. The RNA precipitates were washed with ethanol (CH_3_CH_2_OH, Merck, 75%), dried at room temperature, and dissolved in RNase-free water, and the RNA concentration and purity were detected using a micro UV-visible spectrophotometer (NanoDrop 2000, Thermo Fisher Scientific, USA).
2. mRNA purification and fragmentation: poly(A) mRNA was enriched by oligo(dT)-coupled magnetic beads (mRNA Capture Beads). mRNA was eluted with Tris buffer after two rounds of binding and washing. mRNA fragmentation was performed in a gene amplifier (ProFlex PCR, Thermo Fisher Scientific, USA) with First Strand Synthesis Buffer (Thermo Fisher Scientific, USA) and random primers.
3. cDNA synthesis and library construction: reverse transcriptase was used to synthesize the first strand of cDNA and DNA polymerase I to synthesize the second strand. After magnetic beads purified double-stranded cDNA, it was sequentially subjected to end repair, addition of A-tail, and junction ligation, and uracil-containing junctions were digested using the USER enzyme.
4. PCR amplification and library purification: PCR amplification of DNA from the ligated junction and purification of the amplified product by magnetic beads. Library quality was examined by a fragment analyzer (Qsep400, Bioptic, Changzhou, China) (expected fragment size: 370–470 bp) and a fluorescence quantifier (Qubit4, Thermo Fisher Scientific, USA) (concentration > 1 ng/μL).
5. High-throughput sequencing: Qualified libraries were sequenced on a gene sequencer (Illumina Novaseq6000, Illumina, San Diego, CA, USA) platform.

Ensuring that the sequence reads (Reads) obtained have sufficiently high quality is critical before proceeding to data analysis, as this is fundamental to ensuring the accuracy and reliability of the subsequent analyses. Therefore, strict control and filtering of the raw data were implemented. The specific filtering methods included:

(1) Adapter Removal: Bioinformatics tools and algorithms were employed to accurately identify and remove adapter sequences from the raw data, eliminating interference from these non-target sequences in downstream analyses.
(2) Quality Filtering: The remaining reads underwent quality assessment. Reads containing an excessive proportion of ambiguous bases (N > 10%) were excluded to avoid compromising the accuracy of the analysis. Additionally, a quality threshold was set, and reads were discarded if more than 50% of their bases had a quality score of Q ≤ 10, as such reads are unsuitable for further research.

The quality control processes described above yielded high-quality clean data, which were subsequently used for downstream analyses.

### 2.4. Data Analysis

In this study, differential gene expression analysis was performed using DESeq2_1.48.0 [25] based on raw read counts across all samples. Differentially expressed genes (DEGs) were identified with a threshold of |Fold Change| ≥ 2 and false discovery rate (FDR) < 0.01.

## 3. Results and Discussion

### 3.1. Sequencing Data Quality Control and Results Statistics

Sequencing analysis was performed on five iodine-treated samples to investigate and elucidate the impact of iodine on the growth mechanisms of *Capsicum annuum* leaves at the transcriptomic level. The results obtained (Table 1 and Table 2) are as follows: A total of 39.43 Gb of clean data were generated, with a Q30 value greater than or equal to 94.47%, confirming the reliability of the sequencing quality for the five samples in this study, which meets the requirements for subsequent in-depth analysis. Additionally, when the obtained sequencing data were compared with the gene database of *Capsicum annuum*, the results showed that the transcriptomic sequences of all samples exhibited a high level of specificity, with a mapping rate to the genome reaching 92.87% or higher. This indicates high alignment consistency, providing a solid foundation for further research.

### 3.2. Gene Expression Analysis

The RNA-seq sequencing results are presented in the form of transcript-level reads. Under normal conditions, the expression level of a gene transcript is positively correlated with the number of corresponding reads obtained in the sequencing results. Therefore, the gene expression level can be initially assessed by calculating the number of reads aligned to each transcript (read counts). However, it is important to recognize that the read count values are influenced not only by gene expression levels but also by gene length and sequencing depth. Normalization of mapped reads and transcript length is necessary to reflect transcript expression levels more accurately. In this process, the StringTie tool, using a maximum flow algorithm combined with the FPKM (fragments per kilobase of transcript per million mapped reads) normalization metric, provides a more precise quantification of transcript or gene expression levels.

### 3.3. Differentially Expressed Genes Results Presentation

Before the comparative analysis of the experimental data from these three sample groups, two threshold criteria were set in this study: First, the fold change (FC) in differential gene expression must be greater than or equal to 2, which quantifies the relative change in expression levels between the control and experimental groups. Second, to control for false positive errors introduced by multiple comparisons, the false discovery rate (FDR) adjusted by the significance *p*-value (*p*-value) must be less than 0.01 to ensure the reliability of the conclusions.

**Figure 2:**
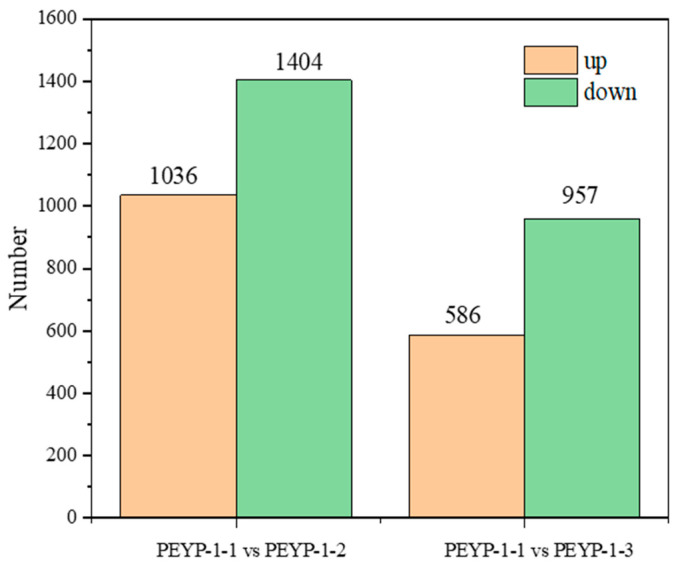
Statistics of upregulated and downregulated differentially expressed genes. Note: The iodine exposure concentrations for the PEYP-1-1, PEYP-1-2, and PEYP-1-3 treatment groups were 2 ppm, 4 ppm, and 8 ppm, respectively. The orange bars represent upregulated genes, while the green bars represent downregulated genes.

This study first screened and quantified the upregulated and downregulated gene expressions. As shown in Figure 2, comparing PEYP-1-1 with PEYP-1-2, a total of 2440 differentially expressed genes were identified, of which 1036 genes were upregulated, and 1404 genes were downregulated. When comparing PEYP-1-1 with PEYP-1-3, 1543 differentially expressed genes were identified, with 586 genes upregulated and 957 genes downregulated.

**Figure 3:**
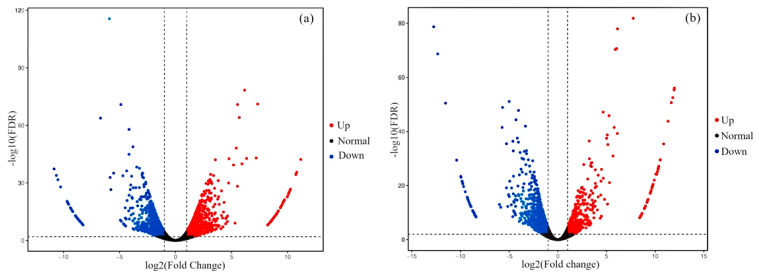
Volcano maps of differential gene expression. Note: (**a**) PEYP-1-1 vs. PEYP-1-2; (**b**) PEYP-1-1 vs. PEYP-1-3. The iodine exposure concentrations for the PEYP-1-1, PEYP-1-2, and PEYP-1-3 treatment groups were 2 ppm, 4 ppm, and 8 ppm, respectively. The horizontal axis represents the log2 (Fold Change), illustrating the difference in fold change, and the vertical axis represents the −log10 (FDR), illustrating the false discovery rate. Among them, the red dot represents the upregulated gene, the gray dot represents the gene with no significant difference, and the blue dot represents the downregulated gene.

Subsequently, volcano plots were generated based on the fold change (FC) and false discovery rate (FDR) of each differentially expressed gene (as shown in Figure 3), aimed at revealing the global distribution characteristics of the differentially expressed genes while highlighting genes that show significant changes under specific thresholds. This provides a foundation for subsequent biological function annotation and pathway enrichment analysis.

### 3.4. GO Functional Enrichment Analysis of Differentially Expressed Genes

To further investigate the toxic effects of different concentrations of iodide exposure on pepper (*Capsicum annuum* L.) leaves, this study first performed Gene Ontology (GO) functional annotation on the differentially expressed genes identified. The analysis of the top 30 significantly enriched GO terms was conducted from three perspectives: Biological Process (BP), Cellular Component (CC), and Molecular Function (MF).

**Figure 4:**
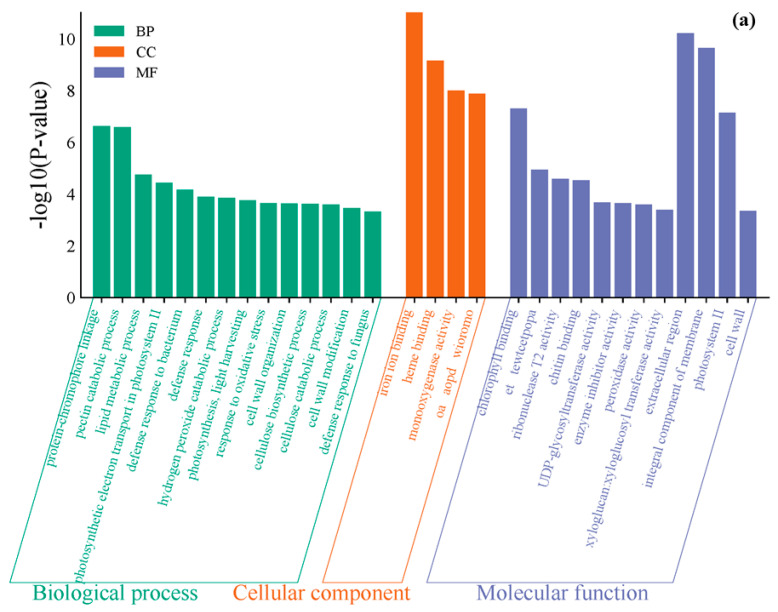

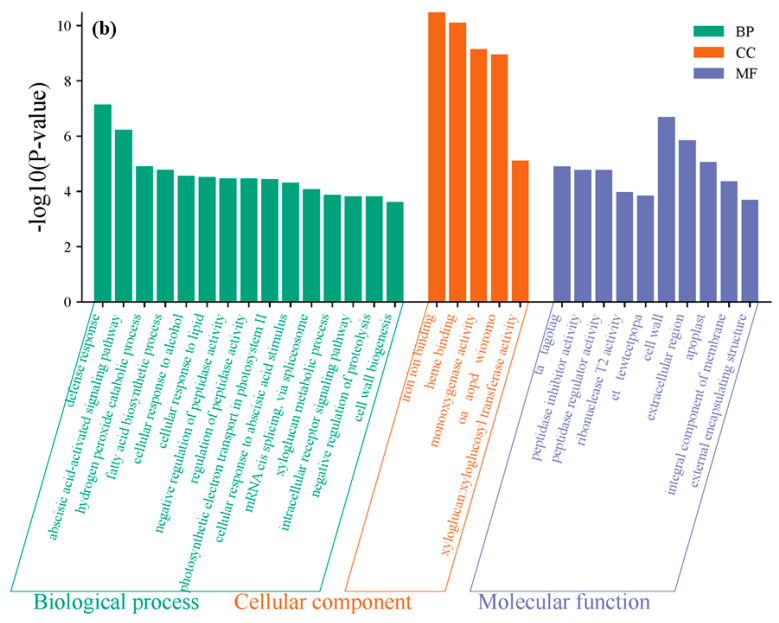
A bar chart of GO functional enrichment analysis for each treatment. Note: (**a**) PEYP-1-1 vs. PEYP-1-2; (**b**) PEYP-1-1 vs. PEYP-1-3. The iodine exposure concentrations for the PEYP-1-1, PEYP-1-2, and PEYP-1-3 treatment groups were 2 ppm, 4 ppm, and 8 ppm, respectively. In the Molecular Function category, OA, AOPD, WIOROMO correspond to oxidoreductase activity, acting on paired donors, with incorporation of or reduction in molecular oxygen, respectively; ET, TEWTCETPOPA correspond to electron transporter and transferring electrons within the cyclic electron transport pathway of photosynthesis activity, respectively; TA and TA, GOTAG correspond to: transferase activity, transferring acyl groups other than amino-acyl groups.

In the GO functional enrichment analysis for PEYP-1-1 vs. PEYP-1-2 (Figure 4a), significant enrichment of GO terms was observed in the molecular function category, specifically in “iron ion binding”. Iron ions play a crucial role in plants by promoting growth, enhancing stress resistance, and participating in photosynthesis, which is essential for plant physiological functions and growth processes.

In the GO functional enrichment analysis for PEYP-1-1 vs. PEYP-1-3 (Figure 4b), significant enrichment of GO terms was also observed in “iron ion binding” under the 8 ppm iodide exposure. Iron ions directly impact key indicators such as plant growth, yield, and stress resistance, making them an important trace element for plant health and development. Iodide ions (I^−^) share similar hydrated ionic radii with iron ions (Fe^2+^/Fe^3+^). This structural similarity may lead to I^−^ competing for the metal coordination sites of iron-binding proteins (ferredoxin or peroxidase) [26], thereby potentially reducing the enrichment of iron ion binding. However, our experimental results show that the enrichment of iron ion binding remains strong under both 4 ppm and 8 ppm iodine stress. This suggests that iodide ions (I^−^) might replace sulfur ligands (cysteine residues) in iron–sulfur clusters rather than directly competing with iron [27], exposing additional iron ion coordination sites and, consequently, enhancing the enrichment of iron ion binding.

### 3.5. KEGG Pathway Enrichment Analysis of Differentially Expressed Genes

#### 3.5.1. Gene Classification Based on Functional Categories

**Figure 5:**
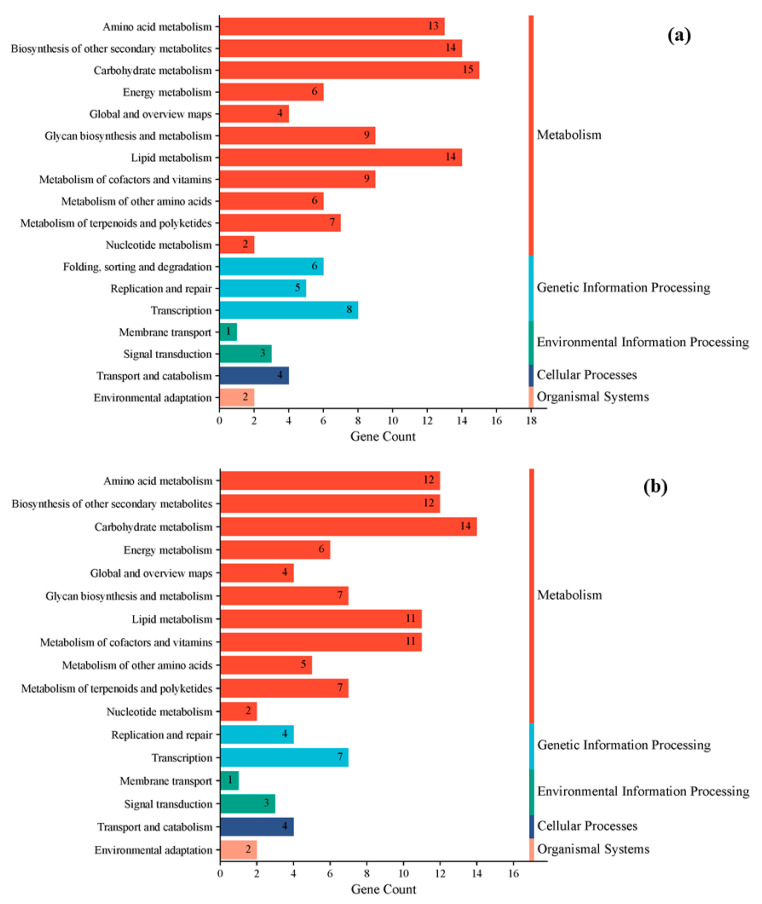
KEGG pathway enrichment classification of genes. Note: (**a**) PEYP-1-1 vs. PEYP-1-2; (**b**) PEYP-1-1 vs. PEYP-1-3. The iodine exposure concentrations for the PEYP-1-1, PEYP-1-2 and PEYP-1-3 treatment groups were 2 ppm, 4 ppm, and 8 ppm, respectively.

In this study, genes were categorized based on their functional groups. As shown in Figure 5, differential genes from all comparisons were predominantly distributed across five major functional categories: Metabolism, Environmental Information Processing, Genetic Information Processing, Cellular Processes, and Organic Systems. Both the PEYP-1-1 vs. PEYP-1-2 and PEYP-1-1 vs. PEYP-1-3 comparisons showed significant enrichment of differentially expressed genes in the Metabolism category, with differential genes accounting for approximately 77% of all genes in these categories.

Further detailed analysis revealed that within the Metabolism category, the biosynthesis of other secondary metabolites and carbohydrate metabolism were the most prominently enriched pathways, with 15 differentially expressed genes identified for each. These results highlight the critical role of metabolic pathways in regulating cellular functions and maintaining tissue homeostasis.

**Figure 6:**
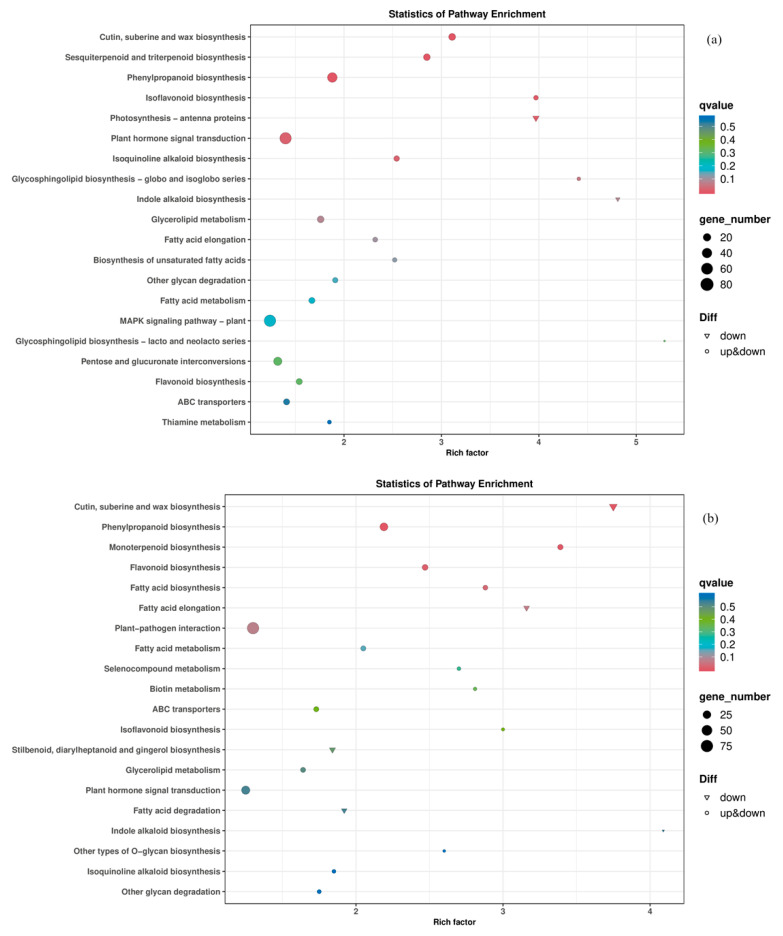
An enrichment bubble map of KEGG pathway for each treatment differential gene. Note: (**a**) PEYP-1-1 vs. PEYP-1-2; (**b**) PEYP-1-1 vs. PEYP-1-3. The iodine exposure concentrations for the PEYP-1-1, PEYP-1-2, and PEYP-1-3 treatment groups were 2 ppm, 4 ppm, and 8 ppm, respectively.

This study performed KEGG pathway enrichment analysis on the differentially expressed genes in pepper leaf samples. Based on gene quantity, enrichment factors, and the significance of Q-value corrections, the KEGG pathway enrichment bubble plots (Figure 6) were used for visualization under two different iodine concentrations.

Under the experimental condition with 4 ppm iodine, the results revealed significant changes in genes associated with the following pathways: cutin, subrine, and wax biosynthesis; sesquiterpenoid and triterpenoid biosynthesis; phenylpropanoid biosynthesis; isoflavonoid biosynthesis; photosynthesis-antenna proteins; plant hormone signal transduction; isoquinoline alkaloid biosynthesis; glycosphingolipid biosynthesis (globo and isoglobo series); indole alkaloid biosynthesis; and glycerolipid metabolism.

Under the experimental condition with 8 ppm iodine, the KEGG pathway enrichment bubble plot (Figure 6b) indicated significant changes in genes involved in the following pathways: cutin, subrine, and wax biosynthesis; phenylpropanoid biosynthesis; monoterpenoid biosynthesis; flavonoid biosynthesis; fatty acid biosynthesis; fatty acid elongation; plant–pathogen interaction; fatty acid metabolism; selenocompound metabolism; and biotin metabolism.

These findings suggest that iodine exposure, particularly at higher concentrations, has a profound impact on various metabolic and signaling pathways in pepper leaves.

#### 3.5.2. The Impact of I^−^ on the Photosynthesis-Antenna Protein Pathway

**Figure 7:**
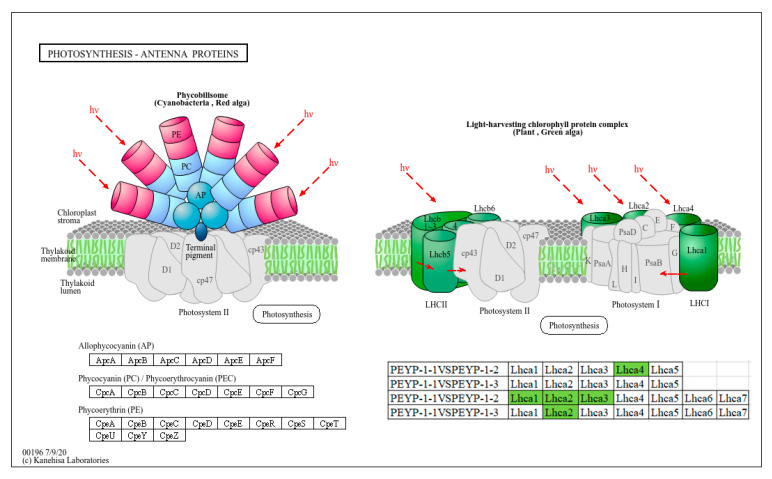
The basic overview of the photosynthesis-antenna protein pathway in PEYP-1-1 vs. PEYP-1-2 and PEYP-1-1 vs. PEYP-1-3. Note: The iodine exposure concentrations for the PEYP-1-1, PEYP-1-2, and PEYP-1-3 treatment groups were 2 ppm, 4 ppm, and 8 ppm, respectively.

In this study, we conducted a detailed analysis of the photosynthesis-antenna protein pathway in pepper leaves exposed to different concentrations of iodine. As shown in Figure 7, under both 4 ppm and 8 ppm I^−^ conditions, no significant changes were observed in the genes associated with the phycobilisome (PBS), a light-harvesting and energy-transfer structure found in algae and certain plants [28]. The stability of the gene expression in PBS indicates that the core process of light pigment energy capture was not significantly impacted by 4 ppm and 8 ppm I^−^. This could be due to the relatively low-stress intensity of I^−^, which did not notably alter the function of PBS. However, under 4 ppm I^−^ treatment, the Lhca4 gene in the light-harvesting complex of photosystem I (LHCI) was downregulated, which may reduce the light energy capture capacity of PSI. This suggests that 4 ppm I^−^ could potentially impair the energy input into PSI, weakening the photochemical reaction efficiency in pepper leaves and affecting the overall function of the electron transport chain [29]. Similarly, three genes (Lhcb1, Lhcb2, and Lhcb3) in the light-harvesting complex of photosystem II (LHCII) were downregulated, which could lead to a decrease in PSII’s light energy utilization efficiency, thus limiting the linear electron flow of PSII [30]. Under the 8 ppm I^−^ treatment, the Lhcb2 gene in LHCII was downregulated. This selective effect might reflect the sensitivity of Lhcb2 to high-concentration I^−^ stress. The downregulation of Lhcb2 reduces the light energy supply to the PSII reaction center, thus weakening the PSII function. However, compared to the 4 ppm I^−^ treatment, Lhcb1 and Lhcb3 were not significantly affected, which could be due to the stronger tolerance of PSI under high I^−^ stress conditions or the preferential protection of PSI by the plant to maintain a certain level of light energy capture capacity.

In plant leaves, to efficiently counteract reactive oxygen species (ROS) generated by light induction, plants rely on specific interactions between LHCI, LHCII, and pigments [11,31]. Through these interactions, plants can efficiently dissipate excess excitation energy as heat, thus enabling a flexible switch between high-efficiency light energy capture and excitation energy release [12,32]. In this physiological process, the differential effects of I^−^ on gene expression in PSI and PSII might reflect different adaptive strategies of pepper plants to stress. At low I^−^ concentrations, the overall inhibition of the photosystem could be a direct effect of stress, whereas at high I^−^ concentrations, the plant may prioritize maintaining PSI function to cope with stronger stress.

#### 3.5.3. The Impact of I^−^ on the Biosynthesis Pathway of Cutin, Suberin, and Wax

**Figure 8:**
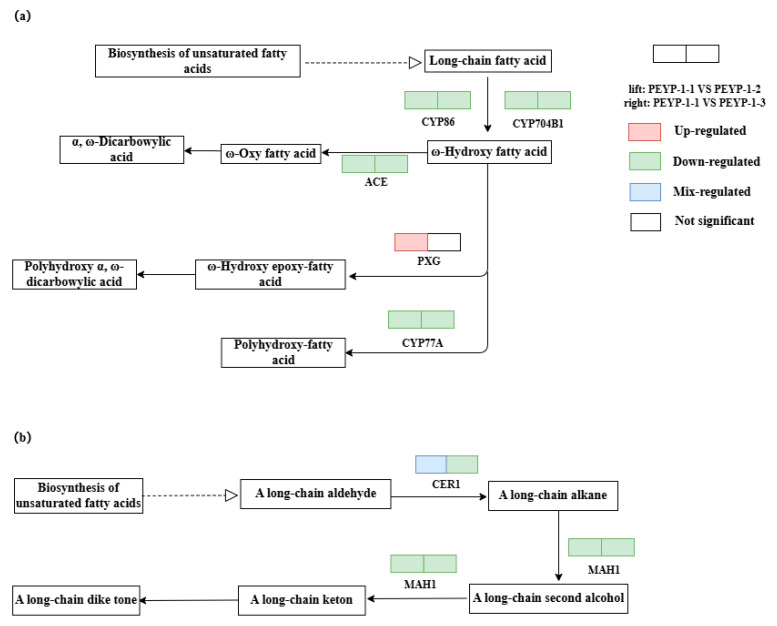
The basic diagram of the biosynthetic pathway of keratin, threonine, and wax. Note: (**a**) Biosynthetic pathway of keratin and threonine; (**b**) wax biosynthetic pathway. PEYP-1-1 was the trace iodine control group of leaves, PEYP-1-2 was the moderate iodine exposure group of leaves, and PEYP-1-3 was the excessive iodine exposure group of leaves.

In this study, we conducted a detailed analysis of the biosynthesis pathways of cutin, suberin, and wax in pepper leaves under iodine exposure. In the biosynthesis pathways of cutin and suberin (Figure 8a), four genes—*CYP86*, *CYP704B1*, *ACE*, and *CYP77A*—were downregulated under both 4 ppm I^−^ and 8 ppm I^−^ conditions. The core function of the *CYP86* subfamily focuses on catalyzing the specific hydroxylation of fatty acyl-CoA molecules at the ω-position, forming ω-hydroxy fatty acids and α, ω-dicarboxylic acids. These processes further contribute to the synthesis of protective biopolymers, particularly suberin and cutin, which are essential protective macromolecules in plants [24,33]. The downregulation of these genes may make plants more susceptible to external adverse factors. Aliphatic dicarboxylic acids, as an important class of organic acids, participate in the acylation of anthocyanins within plants [34,35], forming amphoteric complexes that improve the structural stability and biological activity of plants [36]. Therefore, the downregulation of these four genes decreases the content of dicarboxylic acids in the plant, which, in turn, reduces the adaptability of pepper leaves under stress conditions. Notably, under 4 ppm iodine exposure, the expression of the *PXG* gene showed an upregulation trend. This change promotes the oxidation of ω-hydroxy fatty acids outside the mitochondria, leading to the production of more dicarboxylic acids. This enhanced response may represent a strategy employed by pepper leaf cells to adapt to iodine stress, adjusting metabolic pathways to improve cell adaptability and survival.

In the wax biosynthesis pathway (Figure 8b), the *MAH1* gene was downregulated under both 4 ppm I^−^ and 8 ppm I^−^ conditions. The plant cuticular wax layer plays a crucial role in reducing water loss through transpiration, enhancing plant survival under drought conditions [37], protecting plants from potential UV damage, and defending against pests and diseases [38]. It is mainly composed of a series of very long-chain fatty acids and their derivatives [39], and the *MAH1* gene is involved in the synthesis of long-chain alkanes [35], playing a critical role in wax synthesis. Suppression of this gene expression would make pepper leaves more susceptible to external stress. Notably, under 4 ppm iodine exposure, the expression of the *CER1* gene exhibited both upregulation and downregulation trends, while under 8 ppm iodine exposure, the *CER1* gene showed only a downregulation trend. This suggests that the 8 ppm iodine concentration caused more severe toxic effects on the pepper leaf epidermis. Furthermore, when subjected to iodine stress, it seems that pepper leaves can adjust their epidermal structure to adapt to this unfavorable environment, ensuring the normal growth and development of the plant.

## 4. Conclusions

(1) In this study, 2440 and 1543 differentially expressed genes were identified in pepper leaves under 4 ppm I^−^ and 8 ppm I^−^ conditions, respectively.
(2) Under 4 ppm I^−^ exposure, these genes were significantly enriched in categories such as protein–chromophore linkage, extracellular region function, and iron ion binding in the Gene Ontology (GO) functional enrichment analysis. Under 8 ppm I^−^ exposure, the differentially expressed genes were significantly enriched in categories related to defense response, cell wall components, and iron ion binding.
(3) Under both tested iodine exposure concentrations, Capsicum annuum leaves exhibited significant downregulation of Lhcb2—a key gene encoding the light-harvesting complex II (LHCII) protein in the photosynthesis-antenna pathway (PSII). This suppression impaired photon capture efficiency at the reaction center, thereby reducing photochemical conversion capacity and, ultimately, decreasing overall photosynthetic efficiency. Concurrently, genes involved in cutin, suberin, and wax biosynthesis (*CYP86*, *CYP704B1*, *ACE*, and *CYP77A*) were downregulated. Notably, *CYP86* subfamily enzymes catalyze ω-hydroxylation of fatty acyl-CoA molecules, a critical step in cutin and suberin polymerization. As these hydrophobic polymers constitute essential epidermal barriers, their compromised synthesis likely diminished the plant’s defensive capacity against environmental stressors.

## Figures and Tables

**Table 1:** Original sequence quality control and filtering statistics.

Sample	Clean Reads ^a^	Clean Bases ^b^	Q30 ^c^ (%)	GC Content ^d^ (%)
PEYP-1-1	21,837,760	6,528,919,648	94.90	43.25
PEYP-1-2	23,298,372	6,968,424,342	94.47	43.19
PEYP-1-3	28,962,752	8,655,221,162	94.88	43.31

Note: The total number of pair-end reads in ^a^ clean data; ^b^ clean data: total number of bases; ^c^ clean data: the proportion of bases whose mass value is not less than 30; ^d^ GC content: clean data GC content, that is, the proportion of guanine and cytosine bases in the total base of clean data.

**Table 2:** The statistical table of comparison results between samples and selected reference genome sequences.

Sample	Total Reads Rate ^a^ (%)	Mapped Reads Rate ^b^ (%)	Uniq Mapped Reads Rate ^c^ (%)	Multiple Mapped Reads Rate ^d^ (%)
PEYP-1-1	100	92.87	87.20	5.67
PEYP-1-2	100	93.68	88.34	5.34
PEYP-1-3	100	93.76	87.87	5.90

Note: The total number of ^a^ clean reads, on a single end, is 100%; ^b^ Percentage of reads mapped to reference genome sequences; ^c^ Percentage of reads mapped to specific locations of the reference genome sequence in the total number of mapped reads; ^d^ The percentage of reads mapped to multiple locations in the reference genome sequence out of the total mapped reads.

## Data Availability

Data is contained within the article.
